# Posttranslational modification of Elongation Factor P from *Staphylococcus aureus*

**DOI:** 10.1002/2211-5463.12901

**Published:** 2020-06-16

**Authors:** Alexander Golubev, Luc Negroni, Filipp Krasnovid, Shamil Validov, Gulnara Yusupova, Marat Yusupov, Konstantin Usachev

**Affiliations:** ^1^ Laboratory of Structural Biology Institute of Fundamental Medicine and Biology Kazan Federal University Kazan Russian Federation; ^2^ Département de Biologie et de Génomique Structurales Institut de Génétique et de Biologie Moléculaire et Cellulaire CNRS UMR7104 INSERM U964 Université de Strasbourg Illkirch France; ^3^ Institut de Génétique et de Biologie Moléculaire et Cellulaire CNRS UMR7104 INSERM U964 Université de Strasbourg Illkirch France

**Keywords:** EF‐P, posttranslational modifications, ribosome, stalling, *Staphylococcus aureus*

## Abstract

Antibiotic‐resistant *Staphylococcus aureus* is becoming a major burden on health care systems in many countries, necessitating the identification of new targets for antibiotic development. Elongation Factor P (EF‐P) is a highly conserved elongation protein factor that plays an important role in protein synthesis and bacteria virulence. EF‐P undergoes unique posttranslational modifications in a stepwise manner to function correctly, but experimental information on EF‐P posttranslational modifications is currently lacking for *S*.* aureus*. Here, we expressed EF‐P in *S*.* aureus* to analyze its posttranslational modifications by mass spectrometry and report experimental proof of 5‐aminopentanol modification of *S*.* aureus* EF‐P.

## Graphical abstract

Elongation Factor P (EF‐P) is a conserved protein involved in balance regulation, polyproline motif‐containing proteins, stress resistance and virulence. The interaction between the EF‐P posttranslational modification and the CCA end of the acceptor stem of the initiator tRNA growing peptide allows it to be properly evacuated from the ribosome. We performed mass spectrometry analysis of EF‐P from *S*.* aureus* and identified the presence of 5‐aminopentanolation.

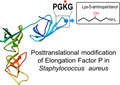

**Abbreviations**
CID: collision‐induced dissociation
EF‐P: Elongation Factor P
HCD: higher‐energy C‐trap dissociation
MS: mass spectrometry
PTM: posttranslational modification
*Sa*EF‐P: EF‐P from *S*.* aureus*

*Staphylococcus aureus* is one of the most common pathogens and a causal agent of health care–associated infections worldwide. Rapidly developing multidrug resistance among staphylococcal clinical isolates urges the search of new antimicrobials against these pathogenic bacteria. Elongation Factor P (EF‐P) is a conserved protein involved in balance regulation of polyproline motif–containing proteins, stress resistance and virulence [1, 2, 3, 4], which makes EF‐P a good candidate to be a target for inhibition of pathogenic bacteria. EF‐P provides specialized translation of proteins with stalling amino acid motif–containing consecutive proline residues (e.g., PPP or APP) [5, 6]. Due to the interaction of the special posttranslational modification (PTM) in the loop in domain I of EF‐P and the CCA end of the acceptor stem, the initiator tRNA‐growing peptide is properly evacuated from ribosome [7]. In contrast with the archaeal/eukaryotic two domain analogues aIF5A and eIF5A, which are uniformly modified with deoxyhypusine moiety [8], three different types of PTM for EF‐P have been revealed in different eubacteria: β‐lysinilation [9], rhamnosilation [10] and 5‐aminopentanolation [11]. Based on the bioinformatical analysis of bacterial genomes [12], it was proposed that bacteria from the genera of *Listeria* and *Staphylococcus* highly likely modify their EF‐P by 5‐aminopentanol, the same type of PTM as for *Bacillus subtilis* EF‐P. The experimental information on the presence and type of EF‐P modification in pathogenic bacteria *S*.* aureus* is currently missing. To define the status of the PTM of EF‐P from *S*.* aureus*, we performed mass spectrometry (MS) analysis of EF‐P from *S*.* aureus* (*Sa*EF‐P) homologically expressed in *S*.* aureus*, and found the presence of 5‐aminopentanolation in the conservative region at K32.

## Materials and methods

### Cloning

For expression of EF‐P tagged with six histidine residues in *S*.* aureus*, the structural gene *efp* fused with histidine tag (*efp*‐his) was subcloned from pET28a:efp into shuttle vector pRMC2 [13]. For this sequence of structural gene coding, EF‐P appended with six codons of histidine and stop codon was amplified using primers pET28aecor1‐f 5′‐TTTTTTGAATTCGTGAGCGGATAACAATTCCCCTCTAG‐3′ and pET28aecor1‐r 5′‐TTTTTTGAATTCATCCTCAGTGGTGGTGGTGGTGG‐3′ using Encyclo polymerase (Eurogene, Moscow, Russia) according to the recommendations of the manufacturer. The resulting fragment containing *efp*‐his was digested with EcoRI (SibEnzyme, Moscow, Russia), purified from agarose gel and ligated with pRMC2 preliminary digested with EcoRI and treated with alkaline phosphatase (SibEnzyme). The ligation mixture was transformed in *Escherichia coli* strain DH5α. DNA from obtained clones was digested with XbaI (SibEnzyme) to reveal constructs with proper orientation of *efp*‐his plasmids. The plasmids resulting in 208‐ and 6877‐bp fragments after treatment with XbaI contain *efp*‐his downstream of Tn‐inducible promoter in the same orientation with the promoter. The resulting plasmid was designated pRMC2:efp‐his. To avoid restriction barriers, we first transformed pRMC2:efp‐his and pRMC2 into *E*.* coli* strain DC10, whose DNA methylation pattern repeats that of *S*.* aureus* [14, 15] . Plasmid DNA isolated from *E*.* coli* strain DC10 was used for electroporation of *S*.* aureus* 6390 using the protocol by Grosser and Richardson [16].

### Protein isolation and purification

For efp‐his expression in *S*.* aureus*, we modified the protocol designed for *E*.* coli* [17]: for induction, oxytetracycline was added to a culture of *S*.* aureus* at concentration 200 ng·mL^−1^. After 4 h of growth in LB medium at 37 °C with agitation at 180 r.p.m., the cells were harvested by centrifugation at 4000 ***g***, washed in cell resuspending buffer and treated with lysostaphin at a concentration of 2 mg·mL^−1^ to lyse the cells. The lysate was cleared using centrifugations at 75 465 ***g*** and then at 234 998 ***g*** for 30 min each. The cleared lysate was applied on Ni‐NTA column (QIAGene, Hilden, Germany). His‐tag‐containing proteins EF‐P and lysostaphin were eluted and separated using anion exchange column MonoQ5/50 GL (GE, Chicago, USA) with column volume of 1 mL using 10 column volumes gradient (0–100% B) with buffers A (10 mm magnesium acetate, 50 mm KCl, 10 mm NH_4_Cl, 5 mm Hepes, pH 7.5, 1 mm DTT) and B (10 mm magnesium acetate, 1 m KCl, 10 mm NH_4_Cl, 5 mm Hepes, pH 7.5, 1 mm DTT) and flow rate of 1 mL·min^−1^. Eluted EF‐P was separated by SDS/PAGE, cut from the gel and analyzed by MS.

### MS analysis

Gel bands were reduced, alkylated and digested with trypsin at 37 °C overnight [18]. Extracted peptides were then analyzed using an Ultimate 3000 nano‐RSLC (Thermo Scientific, San Jose, CA, USA) coupled in line with an Orbitrap ELITE (Thermo Scientific). In brief, peptides were separated on a C18 nanocolumn with a linear gradient of acetonitrile and analyzed in a top 20 collision‐induced dissociation (CID) and a top 10 higher‐energy C‐trap dissociation (HCD) data‐dependent MS. Data were processed by database searching using SequestHT (Thermo Fisher Scientific) with proteome discoverer 2.4 software (Thermo Fisher Scientific) against *S*.* aureus* Swiss‐Prot database. Precursor and fragment mass tolerance were set at 10 p.p.m. and 0.6 Da, respectively, for CID and 10 p.p.m. and 0.02 Da, respectively, for HCD. Trypsin with up to two missed cleavages was set as enzyme. Oxidation (M, +15.995 Da) and 5‐aminopentanol/+101.084 Da (K) were set as variable modification, and carbamidomethylation (C, + 57.021) as fixed modification. Peptides and proteins were filtered with false discovery rate <1%. ProSight Lite was used to confirm the fragment ions assignment [19]. Tables corresponding to the fragments’ masses for the three MS2 spectra are presented in Tables S1–S4.

## Results and Discussion

**Fig. 1:**
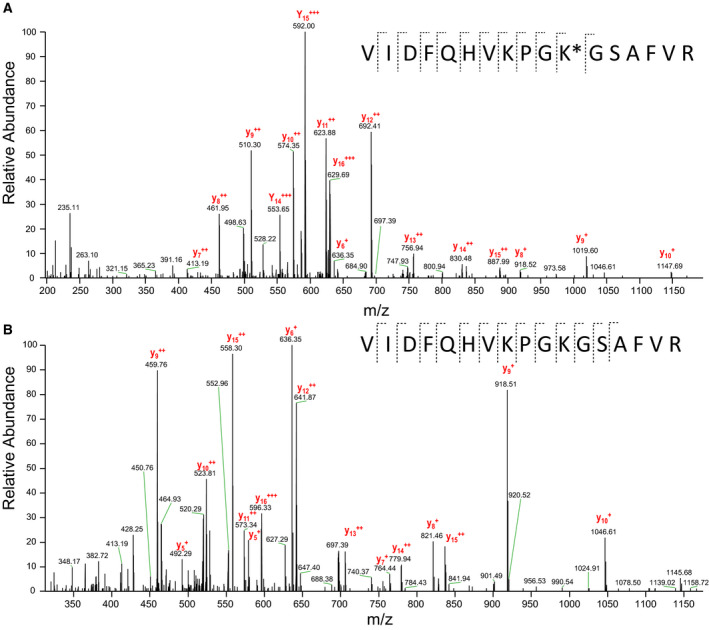
Annotated high‐resolution MS^2^ spectrum for the identification of modification on K32. Observed fragments are indicated on the amino acid sequence and on the high‐resolution Fourier transform‐based mass spectrometry (FT‐MS/MS) spectrum. (A) tandem mass spectrometry (MS/MS) spectrum with 5‐aminopentanol on K32. (B) No modification on K32.

**Fig. 2:**
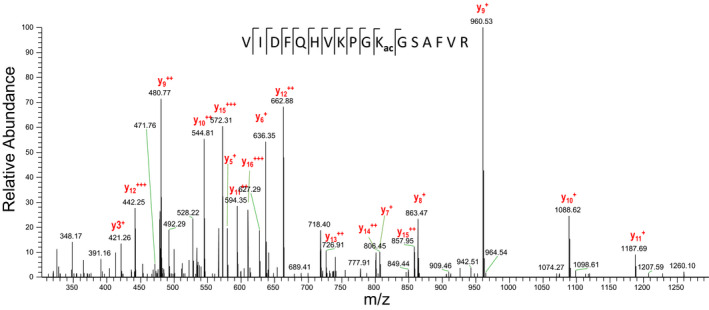
Annotated high‐resolution MS^2^ spectrum for the identification of acetylation modification on K32. Observed fragments are indicated on the amino acid sequence and on the high‐resolution FT‐MS/MS spectrum.

EF‐P is a three‐domain protein that can be found in eubacteria with the exception of at least three species, *Carsonella ruddii*, *Hodgkinia cicadicola* and *Nasuia deltocephalinicola* [20]. It was shown that EF‐P promotes translation of proteins containing polyproline residues and possibly other stalling amino acid motifs [6, 21]. Although the functional analogue of EF‐P–eIF5α is absolutely necessary for the eukaryotic cell, deletion of *efp* gene is not lethal for a number of eubacterial species. This might reflect comparatively rare occurrences of the stalling motifs in these eubacterial species, which makes EF‐P dispensable for basic metabolism of the bacteria [3]. Nevertheless, in some organisms, manifestation of pathogenicity and stress resistance can be remarkably reduced in Δ*efp* strains, for example, in the case of *Salmonella enterica* [2] and *Shigella flexneri* [4]. In *Bacillus subtilis*, loss of EF‐P or its 5‐aminopentanol modification results in a swarming motility defect [11]. Modification of EF‐P was shown to be important for rescuing of ribosome stalling caused by polyproline synthesis. *Pseudohongiella spirulinae*, *Thalassolituus oleivorans* and *Nitrincola nitratireducens* harbor both β‐lysinilation and rhamnosilation systems [22, 23, 24]. The third modification, 5‐aminopentanolation, was found in *B*.* subtilis*, and *Bs*EF‐P has a conservative KPG**K**G motif where the 32nd lysine residue carries the modification [11]. *Sa*EF‐P has a typical three‐domain structure with unstructured loop in domain I and KPG**K**G motif with lysine residue in the 32nd position [17, 25, 26]. To reveal modification in *S*.* aureus*, we carried out homologous expression of *Sa*EF‐P tagged with six histidine residues. Purified protein was used for classical bottom‐up proteomic analysis, a peptide corresponding to sequence (VIDFQHVKPG**K**GSAFVR) was identified with and without 5‐aminopentanol on K32 (Fig. 1A,B). The site specificity was signed by two flanking y fragments (y6 and y7) on either side of lysine K32. We also found an acetylation modification at K32 (Fig. 2). For the three forms of the peptides, the statistics, the ion assignment and the fragment mass error are presented in the Supporting Information (Table S2 for peptide with aminopentanol, Table S3 for nonmodified peptide and Table S4 for acetylated peptide). We assume that the acetylation plays a role as primary group for a 5‐aminopentanol construction, and it is detected during analyses as a stable intermediate of the full modification. However, no valid information about the relative proportion of the different forms of the peptide can be deduced from the signal intensity, considering that a modification on lysine can affect the ionization efficiency. Previously it was shown that only part of EF‐P in bacteria carry modifications [27, 28].

As a result of our experiments, we established that the EF‐P in *S*.* aureus* is modified with 5‐aminopentanol at position K32 within the highly conserved PGKG motif in domain I. The finding makes it possible to apply data about possible enzyme modifiers [27] of this type of EF‐P PTM to the *S*.* aureus* case. When the exact enzyme cascade is established, it will be possible to make an experiment with coexpression of EF‐P with the modifying enzymes, and thus obtain a modified *S*.* aureus* EF‐P in homogeneous form for future structural studies.

Despite the available information, it is still unclear how 5‐aminopentanol is involved structurally in polyproline synthesis. Because of the ambiguity in position of the hydroxyl group [11], the structure of 5‐aminopentanol is not fully clear, and the hydroxyl group could be directly involved in processes of polyproline synthesis. Having fully modified *S*.* aureus* EF‐P in good quantities, by methods of structural biology it will be possible to determine interaction mechanisms of the modification with the ribosome and also determine the exact position of the hydroxyl group in the modification.

**Fig. 3:**
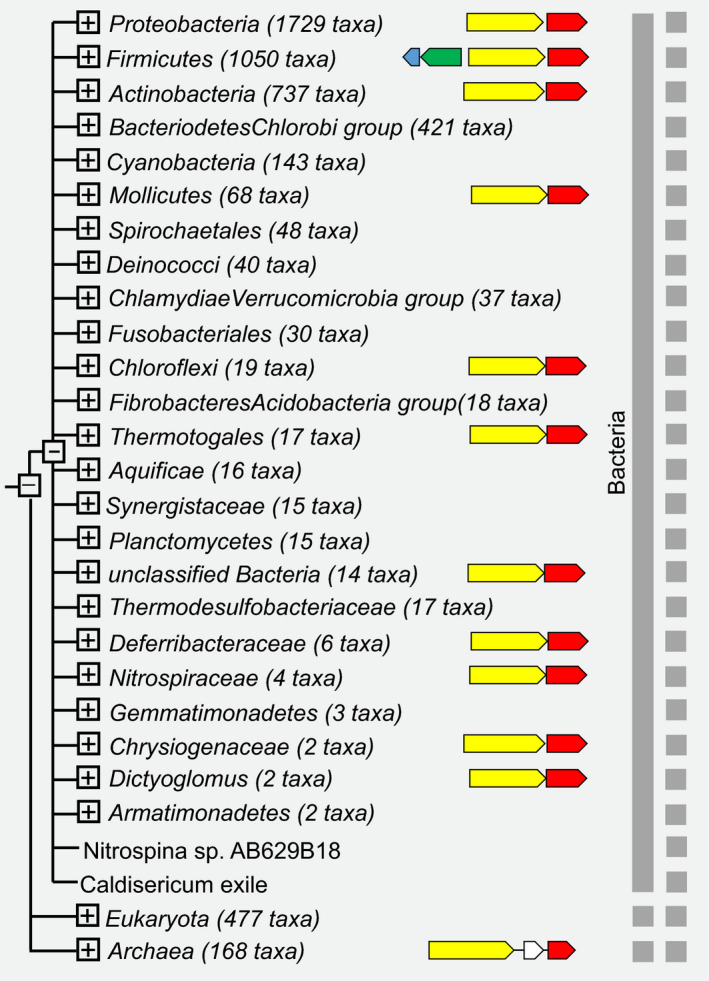
Co‐occurrence of putative proline aminopeptidase (light yellow arrow) and efp (red arrow) revealed by analysis in STRING [29].

Based on ideas that functionally related proteins could colocalize with the EF‐P [9] gene, we checked the EF‐P gene neighborhood using STRING [29] (Fig. 3). We have found that one protein, Xaa‐Pro aminopeptidase, strongly colocalizes within closely related organisms in *Firmicutes*. Taking into account that primary function of EF‐P is the release of the ribosome stalling caused by polyproline motifs, it is highly likely that the proline aminopeptidase could be related to this process role. Supposedly, it could degrade the inactive proteins containing polyproline amino acid motifs, and thus release ribosome from stalling events.

Recently discovered possible enzymes modifiers apparently are involved in the modification process of *S*.* aureus* EF‐P. When the modifying proteins and sequence of modifying EF‐P reactions are clarified, it would be interesting to knock out the enzyme‐modifier genes to check how this affects the functions of EF‐P, fitness of *S*.* aureus* and especially virulence. These studies could show if the modifiers themselves are potential inhibition targets. In the future it would be possible to conduct structural studies of modifier enzymes and their interactions with EF‐P, and find ligands for disruption of these interactions.

In *S*.* aureus* in the part of proteome controlled by EF‐P activity, some proteins are related to virulence [30]. Based on the other studies of bacteria’s EF‐P [2, 4, 11], we can assume that the lack of functional EF‐P, as well as its modifying enzymes, could negatively affect the viability of *S*.* aureus* and possibly lead to a loss of virulence. Thus, further studies in this area can potentially open the way to the creation of new anti‐staphylococcal antibiotics.

## Conclusions

In this study, we report the finding of 5‐aminopentanol PTM at K32 of *S*.* aureus* EF‐P by MS analysis. Using bioinformatic methods, we found putative Xaa‐Pro aminopeptidase colocalized with *S*.* aureus* EF‐P. The Xaa‐Pro aminopeptidase possibly could play a role in ribosome release by means of degradation of stalling peptides. Direct studies of the peptidase could shed light on its exact role. The found PTM opens the way for future studies of the 5‐aminopentanol modification pathway in *S*.* aureus* and studies of the PTM structural details. We hope that this knowledge about the *S*.* aureus* EF‐P modification could kick off research of its modification pathway inhibition. The results obtained could be further used for development of new antistaphylococcal drugs.

## Conflict of interest

The authors declare no conflict of interest.

## Author contributions

AG, GY, MY and KU conceived the project. AG and FK cloned and purified EF‐P protein from *S*.* aureus* under supervision of SV. LN provided MS analysis. AG, LN, SV and KU wrote the manuscript with input from GY and MY. All authors revised the manuscript.

## Supporting information


**Table S1**. Proteome Discoverer output for the MS^2^ spectra corresponding to peptide VIDFQHVKPGKGSAFVR and its modifications.
**Table S2**. HCD MS^2^ scan 1879 corresponding to Fig. 1A, peptide with aminopentanol.
**Table S3**. HCD MS^2^ scan 2197 corresponding to Fig. 1B; nonmodified peptide.
**Table S4**. HCD MS^2^ scan 2780 corresponding to Fig. 2; acetylated peptide.
